# The conservative management for improving Visual Analog Scale (VAS) pain scoring in greater trochanteric pain syndrome: a Bayesian analysis

**DOI:** 10.1186/s12891-023-06443-5

**Published:** 2023-05-26

**Authors:** Yuping He, Yao Lin, Xiaolan He, Chunrong Li, Qingxiu Lu, Junbing He

**Affiliations:** 1 Department of Trauma Sports Orthopedics, Guigang City People’s Hospital, Zhongshan Middle Road 1, Gangbei district, Guigang, Guangxi 537100 PR China; 2 Jieyang Medical Research Center, Jieyang People’s Hospital, Tianfu Road 107, Rongcheng district, Jieyang City, Guangdong 522000 PR China; 3 Department of Orthopedics, People’s Hospital of Guangxi Zhuang Autonomous Region, Nanning, Guangxi PR China

**Keywords:** Greater trochanteric pain syndrome, Conservative management, Visual analogue pain score

## Abstract

**Background:**

Greater trochanteric pain syndrome (GTPS) possesses a harmful influence on quality of life. Numerous conservative management modalities with varying success have been proposed for patients with GTPS. However, it is not clear which treatment is more effective for reducing pain. The purpose of this Bayesian analysis was to assess the current evidence for the effectiveness of conservative treatments on improving Visual Analog Scale (VAS) pain scoring of GTPS and to determine the most effective treatment protocol.

**Methods:**

A comprehensive study search was performed from inception until July 18, 2022, via the electronic databases PubMed, the Cochrane Library, and Web of Science for potential research. The risk of bias assessment for the included studies was independently performed based on the Cochrane Collaboration Risk of Bias Tool. Bayesian analysis was conducted by using ADDIS software (v1.16.5). The DerSimonian-Laird random effects model was used to perform the traditional pairwise meta-analysis.

**Results:**

Eight full-text articles with a total of 596 patients with GTPS were included in the analysis. In comparing ultrasound-guided platelet-rich plasma application (PRP-U) to ultrasound-guided corticosteroid injection (CSI-U), patients who received PRP therapy experienced reduced pain as the VAS decreased significantly (MD, -5.21; 95% CI, -6.24 to -3.64). VAS score in group of extracorporeal shockwave treatment (ESWT) was significant improved than that in exercise (EX) group (MD, -3.17; 95% CI, -4.13 to -2.15). There were no statistically significantly different VAS scores between the CSI-U group and the CSI under landmark (CSI-B) group. The treatment efficacy rankings of the different treatments on improving VAS scores showed that the most likely efficacious treatment was PRP-U (99%) followed by ESWT (81%), CIS-U (58%), usual care (48%), CIS-B (54%), and EX (84%).

**Conclusion:**

Bayesian analysis revealed that PRP injection and ESWT are relatively safe and effective in the treatment of GTPS. More multicenter high-quality randomized clinical trials with large sample sizes are still needed in the future to provide further evidence.

**Supplementary Information:**

The online version contains supplementary material available at 10.1186/s12891-023-06443-5.

## Introduction

Greater trochanteric pain syndrome (GTPS), a recalcitrant lateral painful condition of the articulatory coxae, has a significant negative impact on physical function and sleep quality and affects 1.8 per 1000 patients annually [1]. For many years, this complex condition has been considered to be caused by trochanteric bursitis, with treatments targeting the bursitis [2]. Nevertheless, the bursa tissue from GTPS patients with total hip replacement did not exhibit signs of inflammation [3]. Growing studies have demonstrated the tendinosis or tendon tear of gluteus medius/minimus at the greater trochanter as the most common cause of GTPS rather than the inflammation of mucous bursa or tendon [4, 5]. There is a fascial connection between the thoracolumbar fascia, gluteus maximus, and iliotibial tract, and GTPS is often accompanied by symptoms of radiating pain [6]. Patients with GTPS typically suffer chronic, persistent pain in the posterolateral hip that radiates to the outside of the leg sometimes extending to the knee; the condition may even cause paresthesia of the lower leg and tenderness of the iliotibial band [7, 8]. Reducing pain is particularly important in improving patients' living conditions.

Accurate diagnosis of the specific etiology of GTPS and the degree of gluteal tendon injury are critical to guiding appropriate treatment. Traditional conservative treatments including physical therapy, anti-inflammatory drug treatment and corticosteroid injections (CSIs), are usually used to treat GTPS at the initial stage [9]. In recent times, the use of platelet-rich plasma (PRP) in treating GTPS has become more prevalent because of its efficacy in promoting tissue healing via providing more platelet derived growth factors to the diseased area [10, 11]. Besides, extracorporeal shock wave, a high-energy sound wave that can produce analgesia and nerve ending blocking after acting on the body, has been used successfully for the treatment of musculoskeletal disorders [12]. The therapeutic mechanism of extracorporeal shock wave treatment (ESWT) was involved with several action including anti-apoptosis, anti-inflammation, cartilage protection, inhibition of nociceptors, and tissue and nerve regeneration [13, 14]. Most patients can resolve GTPS by these non-operative treatments with a success rate of more than 90 percent, while only a few refractory cases require surgical intervention [2]. However, there is no defined treatment protocol for GTPS at present, and choosing a more effective treatment for pain relief is of great importance for clinicians [15, 16].

Among the many scales used to assess acute pain, Visual Analog Scale (VAS) pain scoring is one of the most commonly used one-dimensional measurement and evaluation tools of pain intensity in clinical and scientific research of GTPS [17, 18]. The aim of this Bayesian analysis was to assess the effectiveness of conservative management for improvements in the VAS pain scoring in GTPS and to determine the most effective treatment protocol.

## Methods

### Registration and protocol

This study was conducted using Preferred Reporting Items for Systematic Reviews and Meta-analyses (PRISMA) guidelines in accordance with the Cochrane review methodology (Additional file 1) [19]. The protocol was registered with INPLASY (202280068) and the DOI number is https://doi.org/10.37766/inplasy2022.8.0068.

### PICO question

The patient population (P), intervention (I), comparison (C), and outcome (O) framework was used to formulate the research question of our Bayesian analysis as follows:

- ◽ How does conservative management affect the VAS pain score in patients with GTPS?
- ◽ What were the rankings on the effect of conservative management, and which one is more suitable for patients with GTPS?

### Search strategy

A comprehensive, literature search was conducted to identify studies meeting the inclusion criteria within the electronic databases PubMed, the Cochrane Library, and Web of Science from inception until July 18, 2022. Eligible studies were identified with text words or Medical Subject Headings (MeSH): “greater trochanteric pain syndrome,” “GTPS”, “trochanteric bursitis,” or “gluteal tendinopathy”. The search method and details of the query are added in Additional file 2.

### Study selection criteria

The initial eligibility screen of all study types, titles, and abstracts was conducted by two independent authors (YL and YH). Studies that specifically referred to conservative management in patients with GTPS were collected for further assessment. Then, full-text assessments were performed independently, and research achieving the following criteria were candidates for inclusion: 1) randomized controlled trials (RCTs), quasi-RCTs, or clinical controlled trials; 2) patients with GTPS; and 3) VAS pain scoring was included in the outcome measures of the full-text articles. The exclusion criteria were as follows: 1) duplicate studies and 2) animal experimental studies, letters, case reports, review articles, meta-analyses and so on.

### Data collection

Two independent authors (JH and XH) carried out data collection and extraction included first author, year of publication, countries, study type, sample size, age of patients, follow-up time, and the main outcomes from the included full text. For both test and control groups, the values of VAS score which presented as mean ± SD was extracted in to the final Bayesian analysis. Any disagreement concerning study selection and data extraction between the two evaluating authors was resolved through the assessment of discussion or a third author (CS). If we encountered significant skew in the outcome data in the published research, the corresponding author will be contacted to solicit the missing information.

### Assessment of methodological quality

A risk of bias assessment for the included randomized studies was independently conducted by two authors (CL and XL) using the Cochrane Collaboration Risk of Bias Tool [20]. Each author assigned a value of “unclear,” “low”, or “high” bias for each study according to the following characteristics: 1) random sequence generation, 2) allocation concealment, 3) blinding, 4) incomplete outcome data, 5) selective outcome reporting, and 6) other potential threats to validity.

### Statistical analysis

The data were extracted and assessed by using ADDIS software (v1.16.5) and STATA (version 14.0; Stata Corp, College Station, TX, USA). Estimated effects were reported as the mean differences (MDs) with 95% CIs for the continuous outcomes for each study. The DerSimonian-Laird random effects model was used to perform the traditional pairwise meta-analysis [21]. The I^2^ statistic was used to measure the statistical heterogeneity, and moderate-to-high heterogeneity was shown when the value was greater than 50% [22]. To obtain the posterior distributions of model parameters, 20000 burn-ins and a thinning interval of 10 for each chain [200000 iterations (50000 per chain)] were set as 4 different chains of over-dispersed, initial values. The Brooks-Gelman-Rubin method was used to assess the convergence. The Potential Scale Reduction Factor (PSRF) was calculated using this method to compare within-chain and between-chain variance [23]. Outcomes were considered statistically significant when *P* ≤ 0.05.

### Quality of evidence assessment

We assessed the confidence in the evidence based on the GRADE (Grading of Recommendations Assessment, Development, and Evaluation) approach, which classified evidence as very low, low, moderate, or high certainty for each outcome [24].

## Results

### Study selection

**Fig. 1:**
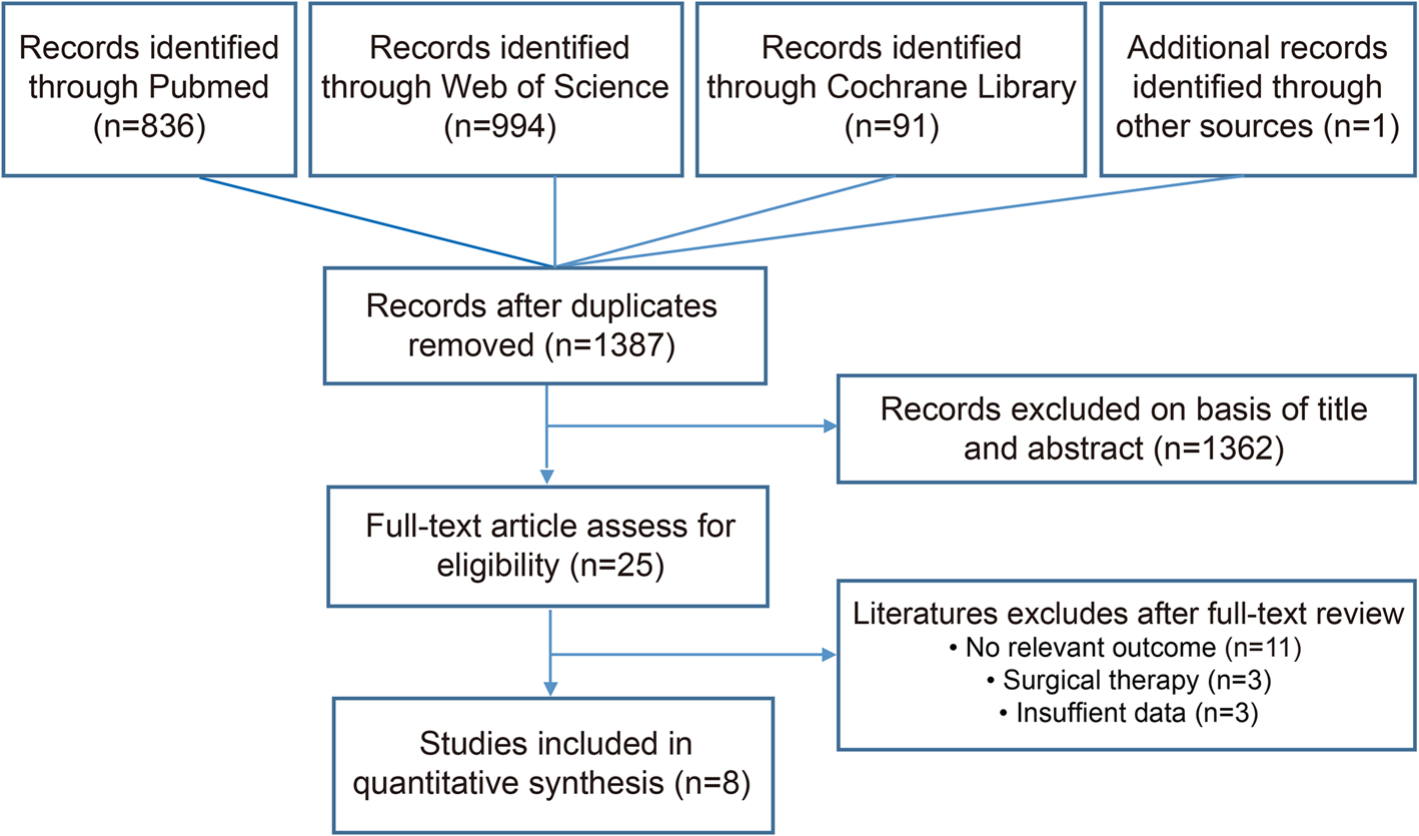
Study inclusion flow chart

Figure 1 shows the flow chart for study inclusion. A total of 836 studies were identified from PubMed; 91 studies were identified from the Cochrane Library; and 994 studies were identified from Web of Science; one additional record was identified through other sources. A total of 1387 publications were found after removing duplicates from the initial search strategy. Of these, after screening the title and abstract, 1362 studies were excluded according to the inclusion and exclusion criteria resulting in 25 studies that underwent full-text assessment. However, 17 of these remaining studies were excluded as no relevant results were provided (11), surgical therapy (3) and insufficient data (3). Finally, 8 studies were enrolled in this Bayesian analysis [25–32].

### Study characteristics

The characteristics of the 8 enrolled studies are presented in Table 1. Among these studies, two studies compared platelet-rich plasma (PRP-U) to glucocorticoid (CSI-U)[25, 26]; three studies compared ESWT to EX [27–29]; one study compared ESWT to CSI-U [30]; one study compared CSI injection under ultrasound guidance (CSI-U) to CSI injection under landmark (CSI-B)[31]; and the remaining one compared CSI-U to usual care (UC)[32]. The total number of participants included in this study was 596, and the sample sizes ranged from 24 to 120 participants with a mean age ranging from 48.7 to 63.73 years. All included studies used the VAS for pain assessment. Follow-up timescales ranged from 24 weeks to 12 months.

**Table 1:** Basic characteristics of the included studies

**Study**	**Country**	**Groups**	**Paintients**	**Age (years)**	**Follow-up**	**VAS (cm)**
Begkas D, 2020 [25]	Athens	T: PRP-U C: CSI-U	24	48.7 (22-79)	24W	T: 1.52 ± 0.505 C: 6.98 ± 0.691
Bashkina A S, 2011 [26]	Russia	T: PRP-U C: CSI-U	40	T: 58.9 ± 7.9 C: 58.2 ± 8.1	6M	T: 0.43 ± 0.69 C: 4.55 ± 2.52
Ramon S, 2020 [27]	Spain	T: ESWT C: EX	100	T: 57.1 ± 12.9 C: 55.6 ± 13	2M	T: 2.0 ± 2.1 C: 4.7 ± 2.1
Furia J P, 2014 [28]	Germany	T: ESWT C: EX	32	T: 51.0 ± 9.9 C: 50.2 ± 14	12M	T: 2.7 ± 0.9 C: 6.3 ± 1.2
Shi L J, 2021 [29]	China	T: ESWT C: EX	53	T: 49.96 ± 6.39 C: 52.79 ± 5.86	6 M	T: 3.20±0.81 C: 6.3 ± 1.4
Heaver C, 2021 [30]	England	T: ESWT C: CSI-U	104	T: 63.73 ± 11.87 C: 60.31 ± 12.74	12M	T: 4.0±2.5 C: 4.82 ± 2.65
Mitchell W G, 2018 [31]	American	T: CSI-U C: CSI-B	30	T: 49.2 ± 12.0 C: 51.5 ± 15.4	6M	T: 1.3 ± 1.9 C: 2.2 ± 2.5
Brinks A, 2011 [32]	Netherlands	T: CSI-U C: UC	120	T: 57.7 ± 13.9 C: 54.8 ± 14.7	12M	At rest T1: 2.1 ± 2.5; C1: 2.3 ± 2.3 With activity T2: 2.8 ± 2.8; C3: 3.2 ± 2.9

*PRP-U* ultrasound-guided platelet-rich plasma application, *CSI-U* ultrasound-guided corticosteroids injection, *ESWT* extracorporeal shockwave treatment, *EX* exercise, *CSI-B* corticosteroids injection under landmark, *UC* usual care

### Risk of bias assessment

**Fig. 2:**
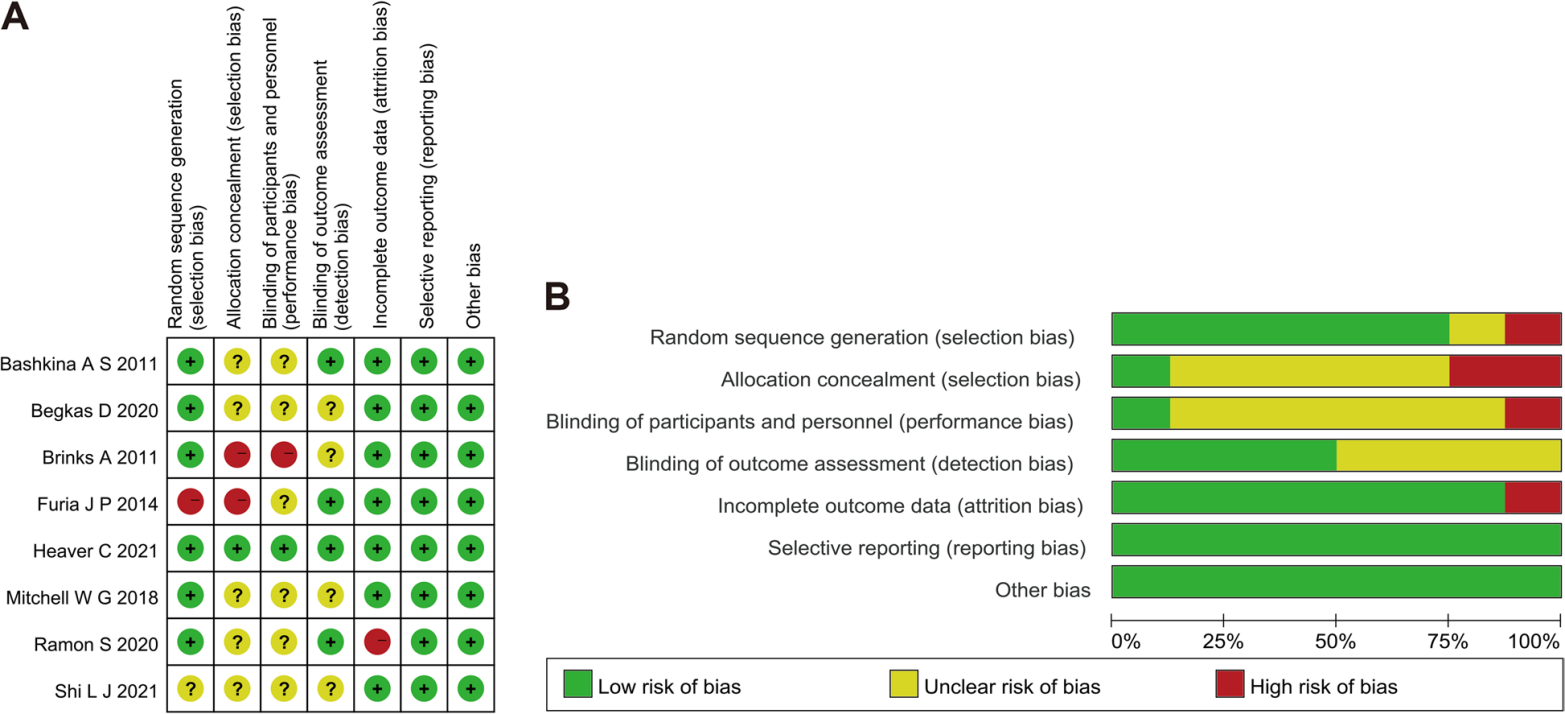
The risk of bias in the included randomized clinical trials (RCTs). **A** Risk of bias summary in RCTs; **B** Risk of bias graph in RCTs

The risk of bias in the included RCTs is presented in Fig. 2A and B, and none of the studies fulfilled all quality indicators. One study was at high risk of selection bias [28]. The lack of reports for allocation concealment [25, 27, 31, 32] and the blinding of participants and personnel [25–29, 31, 32] were the most common, methodological limitations that existed in the enrolled studies [25, 27, 31, 32]. One study had a high risk of incomplete outcomes as more than 10% of patients dropped out during follow-up [27]. Two studies were at high risk of selection bias [26, 28]. Four studies were at high risk of small sample bias due to the small sample size, which was less than 50 [25, 26, 28, 31]. We did not perform a funnel plot since there were fewer than 10 studies in each comparison.

### Visual analog pain score

**Fig. 3:**
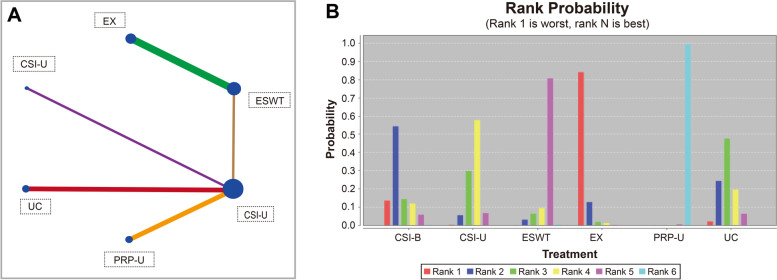
The network map (**A**) and ranking probability (**B**) of the effect of each treatment on Visual Analog Scale (VAS) Pain Scoring

The network map concerning the VAS of the included studies is shown in Fig. 3A. Pairwise random-effects meta-analyses for the VAS are shown in Additional file 3 and Additional file 4. In comparing PRP to corticosteroids, patients in the PRP therapy group experienced pain reduction as the VAS pain scoring decreased significantly (MD, -5.02; 95% CI, -6.25 to -3.79; I^2^=58.3%). In the study in which ESWT was compared with EX, the ESWT group revealed a significant improvement in VAS scores (MD, -3.16; 95% CI, -3.64 to -2.68; I^2^=27%). There were no statistically significantly different VAS scores between the CSI-U- and CSI-B-treated patients (MD, 0.90; 95% CI, -0.69 to 2.49). Another study compared ESWT with CSI-U; although shock wave therapy shows a better tendency to reduce pain, there was no significant difference between the two treatments in improving VAS scores as the confidence interval included zero (MD, -0.82; 95% CI, -1.81 to 0.17). The remaining study [32] that compared CSI and UC did not observe a significant difference in VAS pain scoring improvement (MD, -0.28; 95% CI, -0.94 to 0.37).

The results of the Network Meta-Analysis (NMA) indicated that ESWT interventions markedly elevated the reduction in pain when compared with EX interventions (Table 2; MD, -3.17; 95% CI, -4.13 to -2.15). Most of NMA results was basically consistent with the pairwise random-effects meta-analyses which indicated that it has a certain stability. PRP has obvious advantages in improving VAS scores compared with other treatment groups. The treatment efficacy rankings of the different treatments on improving VAS pain scoring (Fig. 3B and Table 3) showed that the most likely efficacious treatment was PRP-U (99%) followed by ESWT (81%), CIS-U (58%), UC (48%), CIS-B (54%), and EX (84%).

**Table 2:** Network meta-analysis (Consistency model)

**CSI-B**	-0.89 (-3.12, 1.25)	-1.72 (-4.59, 1.09)	1.44 (-1.60, 4.43)	-6.08 (-8.47, -3.38)	-0.59 (-3.16, 1.91)
0.89 (-1.25, 3.12)	**CSI-U**	-0.87 (-2.63, 0.98)	2.30 (0.25, 4.33)	-5.21 (-6.24, -3.64)	0.30 (-0.98, 1.56)
1.72 (-1.09, 4.59)	0.87 (-0.98, 2.63)	**ESWT**	3.17 (2.15, 4.13)	-4.34 (-6.33, -1.92)	1.18 (-1.09, 3.30)
-1.44 (-4.43, 1.60)	-2.30 (-4.33, -0.25)	-3.17 (-4.13, -2.15)	**EX**	-7.50 (-9.70, -4.85)	-2.00 (-4.45, 0.44)
6.08 (3.38, 8.47)	5.21 (3.64, 6.24)	4.34 (1.92, 6.33)	7.50 (4.85, 9.70)	**PRP-U**	5.52 (3.45, 7.06)
0.59 (-1.91, 3.16)	-0.30 (-1.56, 0.98)	-1.18 (-3.30, 1.09)	2.00 (-0.44, 4.45)	-5.52 (-7.06, -3.45)	**UC**

*PRP-U* ultrasound-guided platelet-rich plasma application, *CSI-U* ultrasound-guided corticosteroids injection, *ESWT* extracorporeal shockwave treatment, *EX* exercise, *CSI-B* corticosteroids injection under landmark, *UC* usual care

**Table 3:** Rank probability (rank 1 is worst, rank N is best)

Drug	Rank 1	Rank 2	Rank 3	Rank 4	Rank 5	Rank 6
CSI-B	0.14	0.54	0.14	0.12	0.06	0
CSI-U	0	0.06	0.3	0.58	0.07	0
ESWT	0	0.03	0.06	0.09	0.81	0
EX	0.84	0.13	0.02	0.01	0	0
PRP-U	0	0	0	0	0	0.99
UC	0.02	0.24	0.48	0.2	0.06	0

*PRP-U* ultrasound-guided platelet-rich plasma application, *CSI-U* ultrasound-guided corticosteroids injection; *ESWT* extracorporeal shockwave treatment, *EX* exercise, *CSI-B* corticosteroids injection under landmark, *UC* usual care

### Quality of evidence (GRADE)

In accordance with the GRADE approach, the overall quality of evidence ranged from very low to moderate as shown in Table 4.

**Table 4:** Pair-wise and network meta-analysis estimates of the effects of the main outcomes

Comparison	Number of trials	Pair-wise estimate (VAS, 95% CI)	Quality	NMA estimate (VAS, 95% CI)	Quality
PRP-U vs CSI-U	2	-5.02 (-6.25, -3.79)	Low^a,d^	-5.21 (-6.24, -3.64)	Moderate^a^
ESWT vs EX	3	-3.16 (-3.64, -2.68)	Moderate^b^	-3.17 (-4.13, -2.15)	Low^a,b^
ESWT vs CSI-U	1	-0.82 (-1.81, 0.17)	Very Low^b,c^	-0.87 (-2.63, 0.98)	Very Low^b,c^
CSI-U vs CSI-B	1	-0.90 (-2.49, 0.69)	Low^a,b^	-0.89 (-3.12, 1.25)	Low^a,b^
CSI-U vs UC	1	-0.28 (-0.94, 0.37)	Low^a,b^	-0.30 (-1.56, 0.98)	Low^a,b^

*PRP-U* ultrasound-guided platelet-rich plasma application, *CSI-U* ultrasound-guided corticosteroids injection, *ESWT* extracorporeal shockwave treatment, *EX* exercise, *CSI-B* corticosteroids injection under landmark; ^a^Quality of evidence down by one level for serious imprecision; ^b^Quality of evidence rated down by one level for risk of bias; ^c^Quality of evidence rated down by two levels for very serious imprecision; ^d^Quality of evidence rated down by one level for serious inconsistency

## Discussion

For the management of pain in patients with GTPS, at present, there is no evidence-based approach. Conservative management, the gold standard for the treatment of GTPS, has become more prevalent in recent years [33, 34]. Physical therapy modalities, corticosteroid or local anesthetic injections to the trochanteric area, and shock wave therapy are the commonly used conservative treatment strategies in the clinic. The long-term pain condition is one of the important reasons for the decline in living standards for patients with GTPS. That being said, reducing pain is particularly important for patients with GTPS. It is necessary to conduct a comprehensive and systematic study on the efficacy of conservative treatment in reducing pain, optimizing the treatment plan, and providing better choices for patients. In this Bayesian analysis, we used the accurate and objective VAS as the evaluative index and compared the efficacy of five different treatment schemes in improving patients' pain. Different from the previous situation of combining the pain scores from different pain scales, we excluded studies using other pain scales, such as numerical rating scales [35, 36] and facial expression pain scales [37] and only merged the data from the VAS scale; therein, we ensured the accuracy of the consolidated results as different pain scales have obvious heterogeneity in measurement methods.

Local injection of glucocorticoids is a common method to treat the disease, but its effectiveness, safety, and rationality are controversial. A systematic review of CSI in GTPS has revealed that CSI has a short-term benefit [7]. A previous study considered the precise mechanism of how CSI affects tendon pain; the study concluded this effect on tendon pain was due to its impacts on inflammation and nociception [38]. However, our pairwise random-effects meta-analyses and network analysis demonstrated that CSI had no significant, positive effect on relieving pain when compared with usual care or ESWT. Only in network analysis did the CSI-U produce significant improvement for VAS compared with EX. Recent studies also suggest that the main, pathological change of chronic tendon disease is the degeneration of tendon tissue rather than tendinitis, and there is no acute inflammatory cell infiltration in the lesion [39]. Glucocorticoid injection should be used with caution, and more appropriate treatment options should be sought.

Regenerative injection treatment is another potential therapy option for GTPS. Platelet-rich plasma, an autologous blood product with various anabolic functions for tissue regeneration, is recommended for use in patients with GTPS [25, 26]. The results from our pairwise analysis showed that patients with GTPS under PRP therapy experienced reduced pain as the VAS pain scoring decreased significantly (MD, -5.02). In the field of orthopedic pain management, previous meta-analyses also reported significantly better pain and functional outcomes with PRP treatment versus comparators for lateral epicondylitis [40]. In our network analysis, PRP injection revealed a significant improvement in VAS pain scoring when compared with the other five conservative management approaches for patients with GTPS. In addition, none of the studies reported complications other than pain at the injection site. PRP injections seem to be an effective and safe treatment for GTPS.

In recent years, an accumulating body of research has indicated that ESWT appears to provide beneficial effects in the management of GTPS. According to the study of Carlisi, *et al*., ESWT was efficient in reducing the pain (numeric rating scale) of GTPS at mid-term and short follow-up [41]. Seo, *et al*. retrospectively reviewed the pain and functional outcomes of patients with gluteal tendinopathy and confirmed the positive effect of electrohydraulic ESWT for GTPS as the short-term success rate was 83.3% [42]. In terms of improving the VAS, the results from our pairwise analysis also support this finding. The VAS score was significantly better in patients who accepted the ESWT than in those who exercised (MD, -3.15; 95% CI, -4.10 to -2.17). The shockwaves have both a direct and indirect positive effect on tissues, which may lead to an increase in the release of analgesic substance P, increased neovascularity, and inhibition of COX-II (thereby dampening the inflammatory response) causing overstimulation of nerve fibers and blocking true pain signals through the gate-control theory [30]. On the other hand, our network analysis also found that although both PRP injection therapy and shock wave therapy can bring certain benefits to patients with GTPS, the effect of PRP injection therapy is better than that of shock wave therapy (MD, 4.37; 95% CI, -6.31 to -1.89). In the ranking, PRP injection had the highest probability (99%) of being ranked first as the best treatment followed by ESWT (81%), CSI-U (58%), UC (48%), and CSI-B (54%), and exercise alone had the worst effect (84%). However, there is no direct comparison study between PRP treatment and ESWT, and large-scale, multicenter randomized controlled trials are needed to verify this result in the future.

It is important to note that this study has several limitations. First, the most obvious limitation is the small number of included studies and their small sample sizes, which may have insufficient strength to assess the differences and limited the interpretation of our pooled result. Second, some studies were evaluated using different pain scales, and we were unable to combine all the results. Third, for studies in which this information was shown, there were no differences among studies in the diagnostic process and severity of the disease.

## Conclusions

This Bayesian analysis has supported the efficacy of conservative management in reducing pain in patients with GTPS, and PRP injection therapy has demonstrated superior outcomes for the VAS and has the highest ranking probability as the best treatment for GTPS. ESWT was effective in reducing pain compared with physiotherapy alone. Considering the long-term injury of tendon structure and few GPTS-patients having clinical manifestation of bursitis, we appeal that corticosteroid or anti-inflammatory drug treatment by high-dose or long-term should be avoid. When the initial conservative management including home training, physiotherapy, corticosteroid or anti-inflammatory drug treatment has failed in patients with refractory GTPS, PRP injection and ESWT can serve as viable alternative therapies with safety and efficacy, avoiding unnecessary use of healthcare resources and allowing for cost reduction. We believe that conservative treatments are still the main management of choice for the majority of patients with GTPS in the future, and further research with a high degree of scientific evidence is essential to draw definitive conclusions on optimal conservative management.

## Supplementary Information

**Additional file 1.** 

**Additional file 2.** 

**Additional file 3.** 

**Additional file 4.** 

## Data Availability

All data generated or analyzed during this study are included in this published article and its supplementary information files.

## Abbreviations

GTPS: Greater trochanteric pain syndrome
VAS: Visual Analog Scale
PRP: Platelet-rich plasma application
PRP-U: Ultrasound-guided platelet-rich plasma
CSI-U: Ultrasound-guided corticosteroid injection
EX: Exercise
ESWT: Extracorporeal shockwave treatment
RCT: Randomized controlled trials
PSRF: Potential Scale Reduction Factor
GRADE: Grading of Recommendations Assessment, Development, and Evaluation
NMA: Network Meta- Analysis
