# Supplementary material for: The conservative management for improving Visual Analog Scale (VAS) pain scoring in greater trochanteric pain syndrome: a Bayesian analysis
Source: BMC Musculoskelet Disord. 2023 May 26;24:423. doi: 10.1186/s12891-023-06443-5 (PMC10214555; doi:10.1186/s12891-023-06443-5)
Supplement: Supplementary file 2 — Additional file 2. [file 12891_2023_6443_MOESM2_ESM.doc]

**Additional file 2. Search Strategy.**

**Details of Medline search strategy (searched from inception through July 2022).**

**Search:**((((greater trochanteric bursitis) OR (greater trochanteric pain syndrome)) OR (trochanteric bursitis)) OR (gluteal tendinopathy)) OR (GTPS)

("greater"[All Fields] AND ("trochanteric"[All Fields] OR "trochanteritis"[All Fields]) AND ("bursitis"[MeSH Terms] OR "bursitis"[All Fields] OR "bursitides"[All Fields])) OR ("greater"[All Fields] AND ("trochanteric"[All Fields] OR "trochanteritis"[All Fields]) AND ("somatoform disorders"[MeSH Terms] OR ("somatoform"[All Fields] AND "disorders"[All Fields]) OR "somatoform disorders"[All Fields] OR ("pain"[All Fields] AND "syndrome"[All Fields]) OR "pain syndrome"[All Fields])) OR (("trochanteric"[All Fields] OR "trochanteritis"[All Fields]) AND ("bursitis"[MeSH Terms] OR "bursitis"[All Fields] OR "bursitides"[All Fields])) OR (("gluteal"[All Fields] OR "gluteals"[All Fields]) AND ("tendinopathy"[MeSH Terms] OR "tendinopathy"[All Fields] OR "tendinopathies"[All Fields])) OR "GTPS"[All Fields]

**Translations**

trochanteric: "trochanteric"[All Fields] OR "trochanteritis"[All Fields]

bursitis: "bursitis"[MeSH Terms] OR "bursitis"[All Fields] OR "bursitides"[All Fields]

trochanteric: "trochanteric"[All Fields] OR "trochanteritis"[All Fields]

pain syndrome: "somatoform disorders"[MeSH Terms] OR ("somatoform"[All Fields] AND "disorders"[All Fields]) OR "somatoform disorders"[All Fields] OR ("pain"[All Fields] AND "syndrome"[All Fields]) OR "pain syndrome"[All Fields]

trochanteric: "trochanteric"[All Fields] OR "trochanteritis"[All Fields]

bursitis: "bursitis"[MeSH Terms] OR "bursitis"[All Fields] OR "bursitides"[All Fields]

gluteal: "gluteal"[All Fields] OR "gluteals"[All Fields]

tendinopathy: "tendinopathy"[MeSH Terms] OR "tendinopathy"[All Fields] OR "tendinopathies"[All Fields]

----------------------------------------------------------------------------------------------------------------------

**Details of Web of science search strategy (searched from inception through July 2022).**

**Search:** ((((TS=(greater trochanteric bursitis)) OR TS=(greater trochanteric pain syndrome)) OR TS=(trochanteric bursitis)) OR TS=(gluteal tendinopathy)) OR TS=(GTPS) **Editions:** WOS.SSCI,WOS.AHCI,WOS.CCR,WOS.ISTP,WOS.ESCI,WOS.SCI,WOS.ISSHP,WOS.IC **Timespan:** 1990-01-01 to 2022-07-18 **Results:** 994

----------------------------------------------------------------------------------------------------------------

**Details of Cochrane Library search strategy (searched from inception through August 2021).**

Date Run: 18/07/2022 05:17:37

Comment:

ID Search Hits

#1 (greater trochanteric pain syndrome):ti,ab,kw (Word variations have been searched) 68

#2 trochanteric bursitis 51

#3 gluteal tendinopathy 40

#4 GTPS 62

#5 #1 OR #2 OR #3 OR #4 91
