# Supplementary material for: The conservative management for improving Visual Analog Scale (VAS) pain scoring in greater trochanteric pain syndrome: a Bayesian analysis
Source: BMC Musculoskelet Disord. 2023 May 26;24:423. doi: 10.1186/s12891-023-06443-5 (PMC10214555; doi:10.1186/s12891-023-06443-5)
Supplement: Supplementary file 3 — Additional file 3. [file 12891_2023_6443_MOESM3_ESM.doc]

**
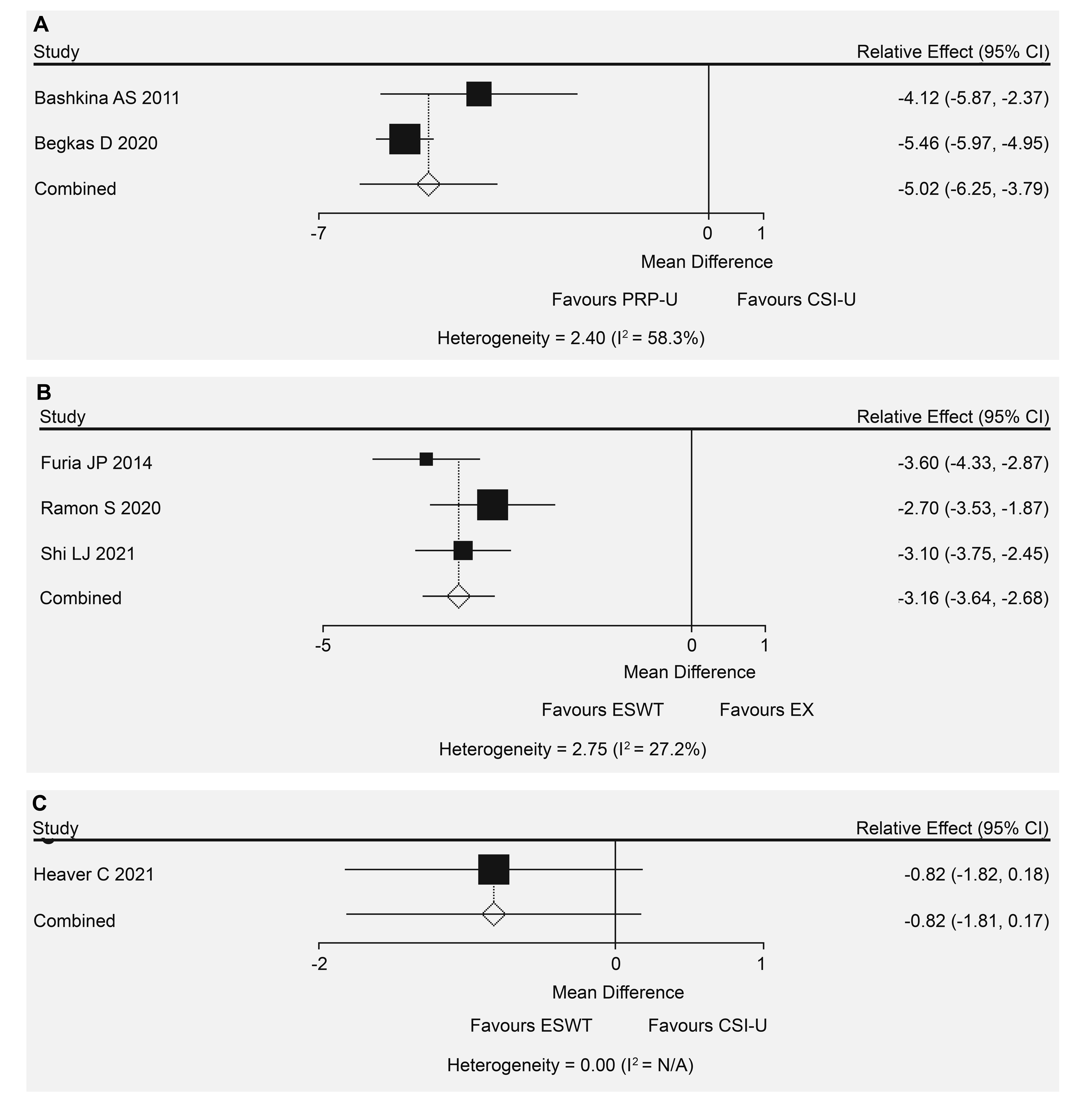
**

**Additional file 3.** Pairwise random-effects meta-analyses for the VAS: PRP-U VS CSI-U (A);

ESWT VS EX (B); ESWT VS CSI-U (C).
