# Supplementary figures and images for: The conservative management for improving Visual Analog Scale (VAS) pain scoring in greater trochanteric pain syndrome: a Bayesian analysis
Source: BMC Musculoskelet Disord. 2023 May 26;24:423. doi: 10.1186/s12891-023-06443-5 (PMC10214555; doi:10.1186/s12891-023-06443-5)

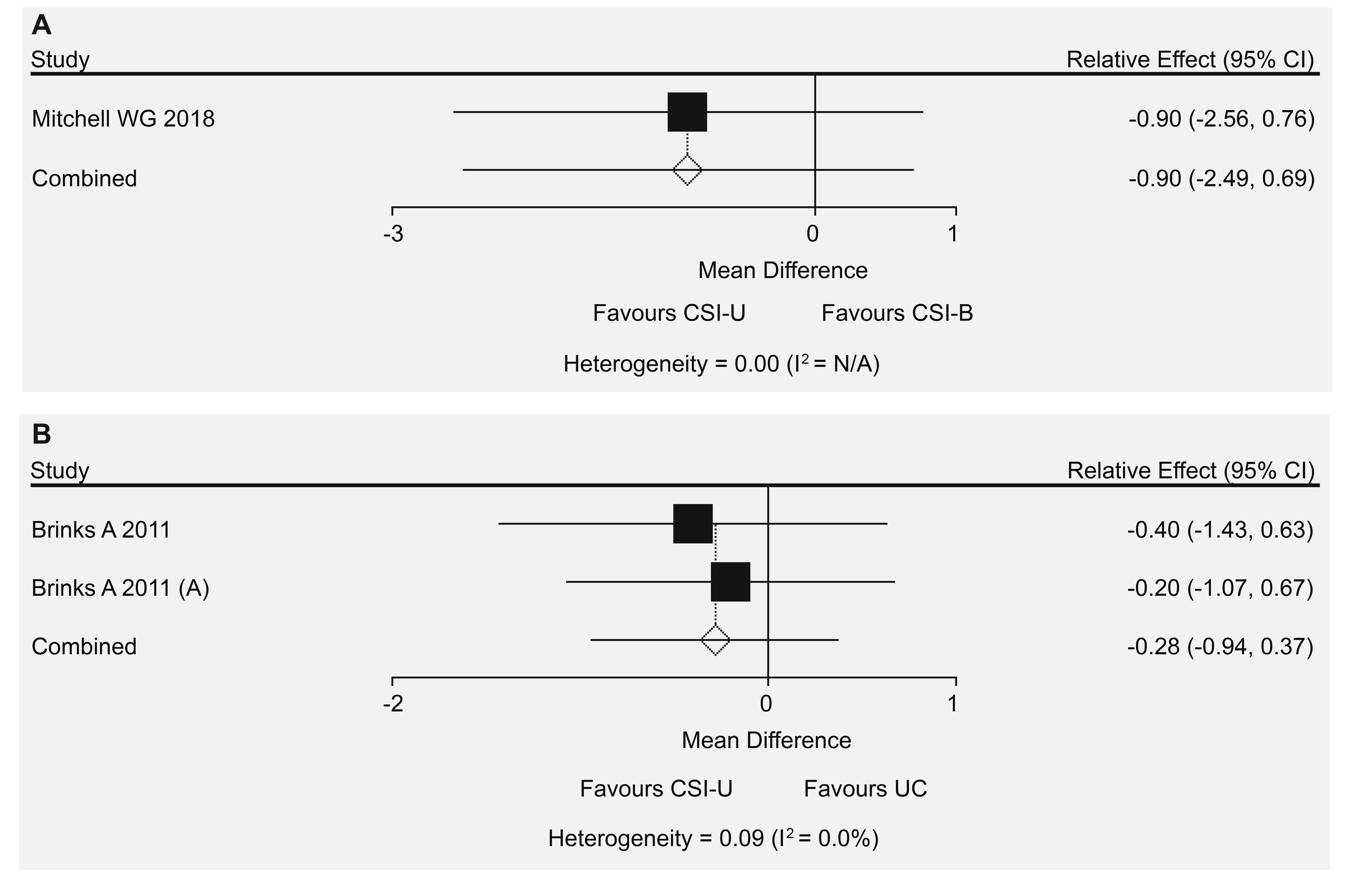


**Additional file 4.** Pairwise random-effects meta-analyses for the VAS: CSI-U VS CSI-B (A);

CSI-U VS UC (B).

Supplement: Supplementary file 4 — Additional file 4. [file 12891_2023_6443_MOESM4_ESM.doc]
